# Effect of Postoperative Radiotherapy for Patients With pIIIA-N2 Non–Small Cell Lung Cancer After Complete Resection and Adjuvant Chemotherapy: The Phase 3 PORT-C Randomized Clinical Trial

**DOI:** 10.1001/jamaoncol.2021.1910

**Published:** 2021-06-24

**Authors:** Zhouguang Hui, Yu Men, Chen Hu, Jingjing Kang, Xin Sun, Nan Bi, Zongmei Zhou, Jun Liang, Jima Lv, Qinfu Feng, Zefen Xiao, Dongfu Chen, Yan Wang, Junling Li, Jie Wang, Shugeng Gao, Luhua Wang, Jie He

**Affiliations:** 1 Department of VIP Medical Services & Department of Radiation Oncology, National Cancer Center/National Clinical Research Center for Cancer/Cancer Hospital, Chinese Academy of Medical Sciences and Peking Union Medical College, Beijing, China; 2 Division of Biostatistics and Bioinformatics, Sidney Kimmel Comprehensive Cancer Center, Johns Hopkins University School of Medicine, Baltimore, Maryland; 3 Department of Radiation Oncology, National Cancer Center/National Clinical Research Center for Cancer/Cancer Hospital, Chinese Academy of Medical Sciences and Peking Union Medical College, Beijing, China; 4 Department of Radiation Oncology, Shanghai Pulmonary Hospital, Tongji University School of Medicine, Shanghai, China; 5 Department of Medical Oncology, National Cancer Center/National Clinical Research Center for Cancer/Cancer Hospital, Chinese Academy of Medical Sciences and Peking Union Medical College, Beijing, China; 6 Department of Thoracic Surgery, National Cancer Center/National Clinical Research Center for Cancer/Cancer Hospital, Chinese Academy of Medical Sciences and Peking Union Medical College, Beijing, China; 7 Department of Radiation Oncology, National Cancer Center/National Clinical Research Center for Cancer/Cancer Hospital & Shenzhen Hospital, Chinese Academy of Medical Sciences & Peking Union Medical College, Beijing & Guangdong, China

## Abstract

**Importance:**

The role of postoperative radiotherapy (PORT) has not been well defined in resected pIIIA-N2 non–small cell lung cancer (NSCLC).

**Objective:**

To evaluate the effect of PORT using modern techniques on survival and safety in patients with pIIIA-N2 NSCLC after complete resection and adjuvant chemotherapy.

**Design, Setting, and Participants:**

The PORT-C randomized clinical trial was conducted in 394 patients with pIIIA-N2 NSCLC treated with complete resection and 4 cycles of platinum-based chemotherapy between January 2009 and December 2017. Data were analyzed between March 2019 and December 2020.

**Interventions:**

Patients were randomized equally into the PORT arm (n = 202) or the observation arm (n = 192). The total dose of PORT was 50 Gy.

**Main Outcomes and Measures:**

The primary end point was disease-free survival (DFS). Secondary end points included overall survival (OS), locoregional recurrence–free survival (LRFS), distant metastasis–free survival, and toxic effects.

**Results:**

In total, 394 patients were enrolled and 364 were eligible, with a median (range) age of 55 (25-70) years. There were 202 (55.5%) male and 162 (44.5%) female patients. The median follow-up was 46.0 (95% CI, 41.9-51.4) months, and 230 DFS events were reported. There were 184 patients in the PORT arm and 180 patients in the observation arm. The 3-year DFS rates were 40.5% with PORT vs 32.7% with observation (median, 22.1 vs 18.6 months), and the difference in DFS was not statistically significant without adjustment (hazard ratio [HR], 0.84; 95% CI, 0.65-1.09; *P* = .20), though it was significant with preplanned yet exploratory analysis (stratified analysis by the number of detected lymph nodes and positive lymph nodes, HR, 0.75; log-rank *P* = .04). The 3-year OS rates were 78.3% vs 82.8% (HR, 1.02; *P* = .93), and LRFS was 66.5% vs 59.7% (HR, 0.71; 95% CI, 0.51-0.97; *P* = .03), respectively. For 310 per-protocol patients (140 with PORT and 170 with observation), PORT significantly improved DFS (42.8% vs 30.6%; HR, 0.75; 95% CI, 0.57-1.00; *P* = .05) but not OS (HR, 0.83; 95% CI, 0.53-1.30; *P* = .41). The 3-year local recurrence only rates were 9.5% and 18.3% in the 2 arms, respectively (Fine-Gray HR, 0.55; Gray test *P* = .04). No radiotherapy-related grade 4 or 5 adverse event was observed.

**Conclusions and Relevance:**

In this phase 3 randomized clinical trial of patients with pIIIA-N2 NSCLC after complete resection and adjuvant chemotherapy, PORT did not improve DFS. Further studies exploring patients who might best benefit from PORT are needed.

**Trial Registration:**

ClinicalTrials.gov Identifier: NCT00880971

## Key Points

**Question:**

Can postoperative radiotherapy (PORT) using modern techniques improve survival of patients with pIIIA-N2 non–small cell lung cancer (NSCLC) after complete resection and adjuvant chemotherapy?

**Findings:**

In this phase 3 randomized clinical trial including 364 eligible patients with pIIIA-N2 NSCLC after complete resection and adjuvant chemotherapy, PORT failed to improve disease-free survival in the intent-to-treat population but significantly improved disease-free survival in the preplanned exploratory stratified analysis and in the per-protocol population. PORT did not improve overall survival.

**Meaning:**

For patients with pIIIA-N2 NSCLC after complete resection and adjuvant chemotherapy, PORT does not improve disease-free or overall survival, and further studies are needed to identify patients who might best benefit from PORT.

## Teaser

This randomized clinical trial evaluates the effect of postoperative radiotherapy on survival and safety in patients with pIIIA-N2 non–small cell lung cancer after complete resection and adjuvant chemotherapy.

## Introduction

A landmark meta-analysis in 1998 showed that postoperative radiotherapy (PORT) was adversely associated with the survival of patients with non–small cell lung cancer (NSCLC).^1^ This was primarily due to the low risk of locoregional recurrence in nonselective patients (including pI-II or pN0-1), high rate of distant metastasis (DM) due to the lack of systemic treatment, and high toxicity due to outdated radiotherapy techniques.^1^ With the rapid improvement of modern radiotherapy techniques and focusing on the subgroup of pN2 NSCLC, there is increasing evidence that PORT results in better survival.^2,3,4,5^ However, the definitive role of PORT in pIIIA-N2 NSCLC, especially in patients receiving adjuvant chemotherapy, remains controversial. To the best of our knowledge, there have been 3 phase 3 randomized clinical trials (RCTs) (Cancer and Leukemia Group B [CALGB] 9734 in North America,^6^ LungART in Europe,^7^ and PORT-C in China). The CALGB 9734 trial was initiated in 1998 and closed in 2000 without meeting the accrual target because of slow accrual^6^; the LungART trial^7^ began in 2007 and completed patient accrual in 2018. The present trial, PORT-C, conducted between 2009 and 2017, was a single-institutional RCT aimed at evaluating the efficacy and safety of PORT using 3-dimensional conformal radiotherapy (3D-CRT)/intensity-modulated radiotherapy (IMRT) in patients with pIIIA-N2 NSCLC after complete resection followed by adjuvant chemotherapy. In this article, we present the results of the PORT-C study, which is the first to complete patient accrual and meets its primary end point among the aforementioned phase 3 RCTs.

## Methods

### Trial Design

The PORT-C RCT was conducted at the National Cancer Center/National Clinical Research Center for Cancer/Cancer Hospital, Chinese Academy of Medical Sciences and Peking Union Medical College in Beijing, China, and approved by the institutional ethics committee. All patients provided written informed consent prior to enrollment. The trial protocol (Supplement 1) was approved by the institutional review board.

### Participants

Patients with histologically confirmed pIIIA-N2 NSCLC (American Joint Committee on Cancer staging system, sixth edition before January 2010 and seventh edition thereafter) who underwent complete resection followed by 4 cycles of adjuvant chemotherapy without recurrence were enrolled. The main eligibility criteria were as follows: age 18 to 70 years, Eastern Cooperative Oncology Group performance status of 0 or 1, less than 10% weight loss before surgery, forced expiratory volume in first second of expiration more than 1 L (or >35% theoretical value, Po_2_ ≥ 70 mm Hg, and Pco_2_ < 45 mm Hg). Patients were excluded if they had pneumonectomy, a history of other cancers, any neoadjuvant chemotherapy, or uncontrolled active infection.

### Randomization

After surgery followed by adjuvant chemotherapy, eligible patients were randomized equally, using simple randomization, to either the PORT or observation arm. Treatment assignment cannot be masked given its open-label design.

### Interventions

Complete resection included lobectomy or bilobectomy with complete exploration and dissection of the mediastinal lymph nodes at levels 4, 7, and 10 for right lung cancer and at levels 4 (if accessible), 5, 6, 7, and 10 for left lung cancer. The location of all the lymph nodes explored was recorded, and the location of all the lymph nodes dissected during the operation was separately noted for pathological examination.

Adjuvant chemotherapy was administered with 4 cycles of platinum-based doublet regimen. Radiotherapy techniques included 3D-CRT and IMRT. The clinical target volume (CTV) included the ipsilateral hilum, subcarinal region, and ipsilateral mediastinum. The stump of the central lesions was also included in the CTV. PORT was administered with 6-MV x-rays at 2 Gy per fraction up to 50 Gy over 5 weeks. Radiation dose constraints for normal tissues were as follows: to the spinal cord, maximum dose less than 45 Gy; volume (V) percentage of organs receiving a specific gray dose: to the heart, V30 less than 40%, V40 less than 30%; and to the whole lung, V20 less than 25% and mean lung dose less than 12 Gy. A central review of each treatment plan was required. Radiotherapy was initiated no later than 6 weeks after the end of adjuvant chemotherapy. The total interruption of radiotherapy for any reason did not exceed 10 days.

### Evaluation and Follow-up

Pre-enrollment evaluation included complete blood cell counts, serum biochemistry, serum tumor markers, electrocardiography, pulmonary function test, computed tomography (CT) or positron emission tomography–CT, brain magnetic resonance imaging or CT (if magnetic resonance imaging is not available), and radionuclide bone scans. All patients were followed up every 3 months for the first 2 years after randomization, every 6 months from years 2 to 5, and yearly thereafter.

### Outcomes

The primary end point was disease-free survival (DFS), defined as the duration between the date of randomization to the date of any disease recurrence or death due to any cause, whichever occurs first. The secondary end points included overall survival (OS; time from the date of randomization to the date of death due to any cause), locoregional recurrence–free survival (LRFS; time from the date of randomization to the date of locoregional recurrence or death, whichever occurs first), DM-free survival (DMFS; time from the date of randomization to the date of DM or death, whichever occurs first), and toxic effects. All time-to-event data, eg, DFS, OS, LRFS, and DMFS, are censored at last follow-up if the corresponding event has not occurred. All adverse events (AEs) were graded using the National Cancer Institute Common Terminology Criteria for Adverse Events, version 3.0.

### Statistical Analysis

Based on the data from our retrospective study,^3^ PORT-C was designed to detect an improvement in 3-year DFS from 30% to 44% (equivalent to an HR of 0.69) at a 1-sided type I error of 0.025 with an 80% power. Assuming a monthly accrual rate of 4.5 patients and guarding against 10% ineligibility or loss to follow-up, the target accrual was 390 patients, and the primary analysis was performed when at least 230 DFS events were observed. The rates of DFS, OS, LRFS, and DMFS were estimated using the Kaplan-Meier method, compared using the log-rank test, and modeled using the Cox proportional hazards method. Analyses of DFS (primary end point) and other time-to-event end points (OS, LRFS, and DMFS) were conducted in a modified intent-to-treat (mITT) basis, eg, all eligible patients were analyzed per randomized assignment, while the per-protocol (PP) population (eligible and randomized participants who adhered to treatment assigned) and the as-treated (AT) population (eligible patients analyzed per treated received) were used as a key sensitivity analysis. As the numbers of detected lymph nodes (DLNs) and positive lymph nodes (PLNs) were deemed important shortly after the study activation,^8^ exploratory stratified analyses, eg, stratified log-rank test and stratified Cox regression model, were also planned in the subsequent protocol amendment, albeit randomization was implemented without these stratification factors. Patterns of the first failures were analyzed using the competing risk analyses, where the cumulative incidences were estimated with the Aalen-Johansen estimator and evaluated using the Gray test and Fine-Gray regression model. Adverse events were summarized among all eligible patients who received PORT (AT population). All statistical tests were at a 2-sided significance level of .05. This report is based on all the data received up to January 31, 2019. All analyses were performed using R, version 3.4.1 (R Foundation).

## Results

### Participants

**Figure 1.**
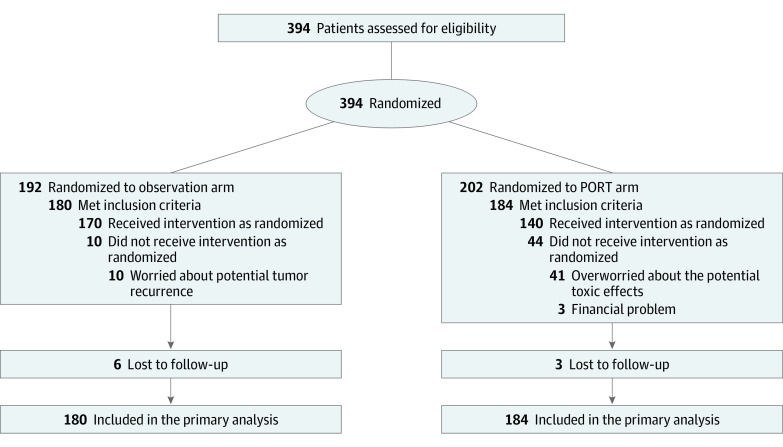
Participant Flow in the Randomized Clinical Trial PORT indicates postoperative radiotherapy.

Between January 1, 2009, and December 31, 2017, 394 patients were enrolled and randomized into the PORT or observation arm. A total of 364 patients were deemed eligible and constituted the mITT population, including 184 patients in the PORT arm and 180 patients in the observation arm (Figure 1 and eAppendix 1 in Supplement 2). The clinical features were well balanced between the 2 arms (Table 1).

**Table 1.** Characteristics of Patients for Modified Intent-to-Treat Analysis

Characteristics	No. (%)		
	Total (n = 364)	PORT (n = 184)	Observation (n = 180)
Gender			
Male	202 (55.5)	108 (58.7)	94 (52.2)
Female	162 (44.5)	76 (41.3)	86 (47.8)
Age, y			
≤60	271 (74.5)	141 (76.6)	130 (72.2)
>60	93 (25.5)	43 (23.4)	50 (27.8)
Median (range)	55 (25-70)	55 (25-70)	55 (32-70)
ECOG PS			
0	177 (48.6)	88 (47.8)	89 (49.4)
1	187 (51.4)	96 (52.2)	91 (50.6)
Smoking history			
Absence	202 (55.5)	94 (51.1)	108 (60.0)
Presence	162 (44.5)	90 (48.9)	72 (40.0)
Tumor location			
Right lung	220 (60.4)	114 (62.0)	106 (58.9)
Left lung	144 (39.6)	70 (38.0)	74 (41.1)
cN2			
No	211 (58.0)	101 (54.9)	110 (61.1)
Yes	144 (39.4)	80 (43.5)	64 (35.6)
Unknown	9 (2.5)	3 (1.6)	6 (3.3)
Pathology			
Non-SCC	305 (83.8)	155 (86.1)	150 (81.5)
SCC	59 (16.2)	25 (13.9)	34 (18.5)
Tumor size			
≤3 cm	190 (52.2)	92 (50.0)	98 (54.4)
>3 cm	174 (47.8)	92 (50.0)	82 (45.6)
Visceral pleura			
Positive	241 (66.2)	123 (66.8)	118 (65.6)
Negative	123 (33.8)	61 (33.2)	62 (34.4)
pT			
T1	81 (22.3)	40 (21.7)	41 (22.8)
T2-3	283 (77.7)	144 (78.3)	139 (77.2)
DLNs			
≤20	172 (47.3)	96 (52.2)	76 (42.2)
>20	192 (52.7)	88 (47.8)	104 (57.8)
PLNs			
1-3	153 (42.0)	82 (45.6)	71 (38.6)
≥4	211 (58.0)	113 (61.4)	98 (54.4)
Positive N2 nodes, median	2 (1-20)	2 (1-17)	2 (1-20)

Abbreviations: DLN, detected lymph node; ECOG PS, Eastern Cooperative Oncology Group performances status; PLN, positive lymph node; PORT, postoperative radiotherapy; SCC, squamous cell carcinoma.

### Adherence

Adherence with treatment assignments was assessed using protocol treatment reviews. In the PORT arm, 44 patients (23.9%) refused PORT; 140 patients (76.1%) were protocol adherent, and all completed PORT, including 125 patients (89.3%) receiving IMRT and 15 patients (10.7%) receiving 3D-CRT. In the observation arm, 10 patients (5.6%) received PORT, and 170 (94.4%) were protocol adherent. Thus, 310 patients were suitable for PP analysis.

### Outcomes

**Figure 2.**
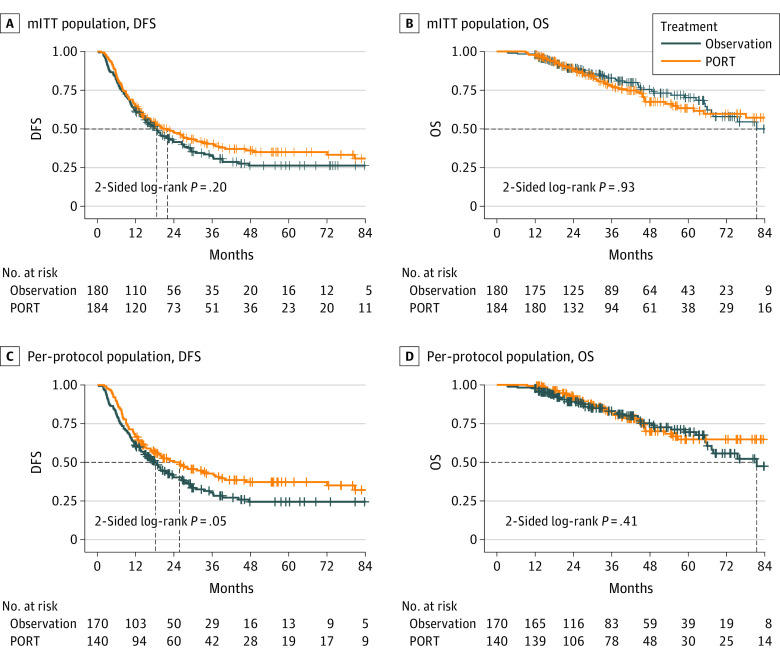
Kaplan-Meier Curves by Arm for Survivals Using Modified Intent-to-Treat (mITT) and Per-Protocol Populations A, Disease-free survival (DFS) of mITT analysis; B, Overall survival (OS) of mITT analysis; C, DFS of per-protocol analysis; D, OS of per-protocol analysis. PORT indicates postoperative radiotherapy.

At the time of this analysis, 230 DFS events were reported, and the median follow-up time was 46.0 (95% CI, 41.9-51.4) months. For mITT analysis, the median DFS for patients in the PORT arm and observation arm was 22.1 (95% CI, 14.8-29.3) months and 18.6 (95% CI, 14.3-23.0) months, respectively. The 3-year DFS was 40.5% and 32.7%, respectively. There was no significant difference in DFS between the 2 arms in the unadjusted analysis (hazard ratio [HR], 0.84; 95% CI, 0.65-1.09; 2-sided log-rank *P* = .20; Figure 2A, Table 2). However, in a preplanned yet exploratory analysis, the DFS significantly differed after stratification according to the number of DLNs and PLNs (HR, 0.75; 95% CI, 0.58-0.98; 2-sided log-rank *P* = .04).

**Table 2.** Summary of Efficacy Results

Outcome	mITT analysis		PP analysis		AT analysis	
	HR (95% CI)	*P* value	HR (95% CI)	*P* value	HR (95% CI)	*P* value
DFS	0.84 (0.65-1.09)	.20	0.75 (0.57-1.00)	.05	0.73 (0.56-0.96)	.02
OS	1.02 (0.68-1.52)	.93	0.83 (0.53-1.30)	.41	0.72 (0.48-1.09)	.12
LRFS	0.71 (0.51-0.97)	.03	0.56 (0.39-0.80)	.002	0.52 (0.37-0.74)	<.001
DMFS	0.94 (0.72-1.22)	.62	0.85 (0.63-1.14)	.28	0.82 (0.62-1.08)	.15

Abbreviations: AT, as-treated; DFS, disease-free survival; DMFS, distant metastasis–free survival; HR, hazard ratio; LRFS, locoregional recurrence–free survival; mITT, modified intent-to-treat; OS, overall survival; PP, per-protocol.

A total of 97 deaths were reported. The median OS was not reached in the PORT arm and 81.5 (95% CI, 61.6-101.4) months in the observation arm. The 3-year OS rates were 78.3% and 82.8%, respectively (unadjusted HR, 1.02; 95% CI, 0.68-1.52; 2-sided log-rank *P* = .93; stratified HR, 0.92; 95% CI, 0.61-1.39; 2-sided log-rank *P* = .70; Figure 2B, Table 2). The 3-year LRFS rates were 66.5% and 59.7%, respectively, with a significant difference between the 2 groups (HR, 0.71; 95% CI, 0.51-0.97; 2-sided *P* = .03; Table 2). The 3-year DMFS rates were 42.0% and 38.2%, respectively (HR, 0.94; 95% CI, 0.72-1.22; 2-sided *P* = .62; Table 2).

**Figure 3.**
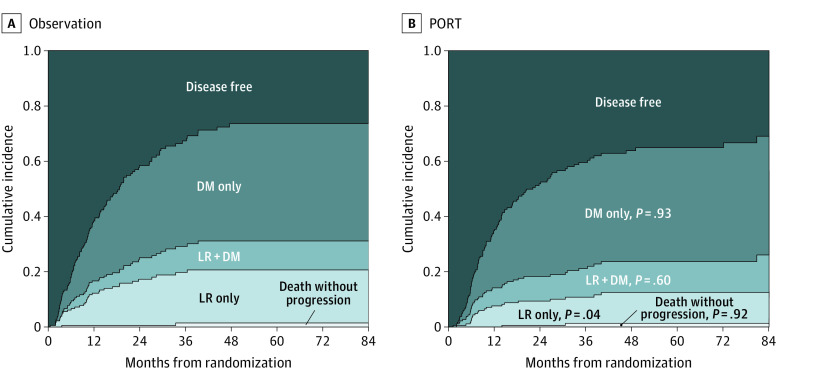
Failure Pattern, Modified Intent-to-Treat Population DM indicates distant metastasis; LR, locoregional recurrence; PORT, postoperative radiotherapy.

Treatment failures of any type were observed in 226 patients, including 110 (59.8%) and 116 (64.4%) in the PORT and observation arms, respectively. Among the 87 patients with first failure of locoregional recurrences (LRs) (including 36 patients with both LR and DM), 39 and 48 patients belonged to the PORT and observation arms, respectively. The 3-year LR only (eg, not including concurrent LR and DM) rates were 9.5% and 18.3% in the 2 arms, respectively (Fine-Gray HR, 0.55; 95% CI, 0.31-0.97; Gray test *P* = .04; Figure 3). Among the 175 patients with first failure of DMs (including 36 patients with both LR and DM), 91 and 84 patients belonged to the 2 arms, respectively. The 3-year DM only (eg, not including concurrent LR and DM) rates were 38.4% and 38.1%, respectively (Fine-Gray HR, 1.00; 95% CI, 0.72-1.38; Gray test *P* = .93).

As a key sensitivity analysis, the PP population consisted of 310 patients, including 140 and 170 patients in the PORT and observation arms, respectively. PORT significantly improved the DFS (3-year: 42.8% vs 30.6%; HR, 0.75; 95% CI, 0.57-1.00; 2-sided *P* = .05; Figure 2C, Table 2) and LRFS (3-year: 71.9% vs 58.4%; HR, 0.56; 95% CI, 0.39-0.80; 2-sided *P* = .002, Table 2), but not OS (3-year: 82.6% vs 83.1%; HR, 0.83; 95% CI, 0.53-1.30; *P* = .41; Figure 2D, Table 2) or DMFS (3-year: 43.6% vs 36.4%; HR, 0.85; 95% CI, 0.63-1.14; *P* = .28; Table 2). As-treated analysis results are also provided in Table 2.

### Toxic Effects and Cause of Death

No radiotherapy-related grade 4 or 5 AEs were observed. Grade 2 or lower radiation esophagitis and radiation pneumonitis were observed in 55 (36.6%; grade 1: 27.3%, and grade 2: 9.3%) and 20 (13.3%; grade 1: 8%, and grade 2: 5.3%) patients, respectively. Only 1 patient (0.7%) had grade 3 radiation pneumonitis. No grade 3 radiation esophagitis was observed.

A total of 97 deaths occurred up to the last follow-up; of these, 47 of 50 deaths (94.0%) in the PORT arm and 42 of 47 deaths (89.4%) in the observation arm were due to cancer progression. Among the 8 non–cancer-related deaths, 1 was due to a second cancer; 3, cardiopulmonary disease; 2, drug AEs; 1, suicide because of depression; and 1, unknown cause.

## Discussion

There is no high-level evidence supporting the benefit of PORT with 3D-CRT/IMRT after complete resection followed by adjuvant chemotherapy for patients with pIIIA-N2 NSCLC. In this PORT-C study, PORT did not prolong DFS in the unadjusted analysis in the mITT population. To the best of our knowledge, this is the first phase 3 RCT to complete patient accrual and meet its primary end point.

However, at the time of study activation, the roles of DLNs and PLNs were not fully recognized, and simple randomization was used. As such, besides unadjusted analysis per randomization, we also conducted preplanned stratified analyses based on the mITT population to provide full information, which showed that PORT significantly improved DFS. Further studies exploring which patients will optimally benefit from PORT are required.

In the PORT-C study, PORT did not significantly improve OS in the mITT population (unadjusted HR: 1.02; 95% CI, 0.68-1.52), PP population (unadjusted HR, 0.83; 95% CI, 0.53-1.30), or AT population (unadjusted HR, 0.72; 95% CI, 0.48-1.09; Table 2). Notably, the study was not designed to detect clinically meaningful differences, had they existed. In fact, despite a total of 97 deaths, there was only 15% and 33% power to detect the effects observed in the PP and AT populations, respectively.

Most recently, the LungART study^7^ showed that PORT did not significantly improve DFS (3-year DFS: 47.1% vs 43.8%; *P* = .16) or OS (3-year: 66.5% vs 68.5%; HR not provided yet). Combined with the results from the 2 phase 3 RCTs, we do not recommend PORT for patients with pIIIA-N2 NSCLC after complete resection followed by adjuvant chemotherapy.

Our study showed that PORT is safe and well tolerated. None of the 150 patients (140 in the PORT arm and 10 in the observation arm) who actually received PORT developed grade 4 or higher AEs. Only 1 patient (0.7%) had grade 3 radiation pneumonitis. Such low toxic effects are largely due to modern radiation techniques, including 3D-CRT and IMRT. In addition, the incidence rates of grade 2 or higher radiation pneumonitis (6%) and grade 3 or lower radiation esophagitis (36.6%) in our study were also lower than the previously reported rates of 50.7% for grade 3 or lower radiation esophagitis^9^ and of 19% for grade 2 or higher radiation pneumonitis.^10^ This may be mainly due to the majority of patients in the present study receiving IMRT (n = 134, 89.3%) rather than 3D-CRT. Another explanation for the low rate of toxic effects is the markedly tighter dose restrictions to the organs at risk in our study. For example, the lung V20 was limited to less than 20%, mean lung dose less than 12 Gy, heart V30 less than 40%, and heart V40 less than 30%. The actual median mean lung dose and V20 were only 9.63 Gy and 16.73%, respectively (eAppendix 2 in Supplement 2). In addition, the CTV in our study did not include the contralateral mediastinum or supraclavicular region, which effectively lowered the exposure dose to the organs at risk, especially the lung and heart. The lower prescription dose of 50 Gy also contributed to the low rate of toxic effects. Corso et al^11^ found that patients with N2 disease who received PORT of 45 to 54 Gy had superior 5-year OS relative to patients who did not undergo PORT (38% vs 27.8%; *P* < .001), but this advantage disappeared if the dose was greater than 54 Gy. Karakoyun-Celik et al^12^ also demonstrated that PORT greater than 54 Gy was associated with poor survival due to an increase in cardiac toxic effects.

In addition, PORT with 3D-CRT/IMRT can guarantee sufficient irradiation doses to the target volume, which is helpful for locoregional control. In the present study, PORT significantly improved LRFS in the mITT population. Similar results were also observed in many other studies,^3,13,14^ which confirmed the advantage of PORT in improving locoregional control. Competing risk analysis of first failure patterns further revealed that the benefit in improving DFS is largely due to locoregional control.

Patients with resected pN2 NSCLC, having a high risk of LR, are believed to benefit from PORT.^5^ Recently published data show a high rate of LR in pN2 NSCLC after surgery, ranging from 35% to 60%.^3,6,14,15^ Thus, several retrospective studies focusing on the pN2 cohort reported that PORT could be associated with improvement in both locoregional control and survival.^3,11,16^ In addition, 2 updated meta-analyses consistently reported that PORT could be associated with improvement in survival in N2 NSCLC.^17,18^ However, in the present study, PORT did not significantly improve the DFS or OS of patients with pIIIA-N2 NSCLC in the unstratified mITT population. This negative result conforms with the findings of the LungART study,^7^ although both RCTs confirmed that PORT was effective in reducing LR. As N2 NSCLC is a heterogeneous group of diseases, some, but not all, patients could benefit from PORT.^8,19^ Further studies are needed to accurately identify the appropriate patients who will optimally benefit from PORT, by using more detailed clinical features and molecular genetics information.

Adjuvant chemotherapy is now the standard of care for patients with resected node-positive NSCLC. In the present study, only patients who completed 4 cycles of adjuvant chemotherapy were included. Effective systemic therapy is crucial to translate locoregional benefits from PORT into improved survival by reducing distant relapse. Recently published studies based on the National Cancer Database revealed that PORT could significantly improve local control and survival for N2 NSCLC treated with adjuvant or neoadjuvant chemotherapy.^16,20^ However, even with requisite adjuvant chemotherapy, our study still showed a high rate of DM (61.9%) and thus no benefits on DFS (40.5% vs 32.7%; *P* = .20) or OS (78.3% vs 82.8%; *P* = .93). Similar results were observed in the LungART study,^7^ which enrolled patients receiving neoadjuvant and/or adjuvant chemotherapy. The study showed that nearly 75% of DFS events were DM, and PORT could not improve DFS or OS. The results of the 2 RCTs suggest the need for further strengthening systemic therapy or finding more effective alternative agents.

The OS rate in the observation arm was higher than expected and comparable to that of the PORT arm. This may be due to a successful follow-up strategy and effective salvage treatments. All enrolled patients were followed up strictly according to the design, which achieved early detection of tumor relapse and timely salvage treatment. The effectiveness of intensive follow-up and its positive association with survival were confirmed in a recently published study.^21^ For patients who experienced locoregional recurrence after resection, salvage radiotherapy could be well tolerated and yield an encouraging outcome.^22^ Before the subgroup of patients fit for PORT can be precisely selected, the strategy of strict follow-up and salvage treatments including salvage radiotherapy for recurrence is clinically feasible.

### Limitations

The most significant limitation of this study was the relatively poor adherence of patients in the mITT population after randomization. In total, 44 of 184 patients (21.7%) in the PORT arm refused PORT, and 10 of 180 patients (5.6%) in the observation arm actually received PORT. This undoubtedly made it more difficult to detect the DFS benefit from PORT in the mITT analyses. Another limitation was that the study was conducted in a single center. Single-center studies are known to have limited generalizability and robustness to support widespread clinical practice.^23^ For example, a relatively lower percentage of patients in the present study (44.5%) had a smoking history compared with a published study (60.6%),^24^ which may be due to a relatively high percentage of adenocarcinoma (80.5%) and female patients (44.5%) in our study. The results need external validation. Finally, treatment modalities have improved during the long enrollment interval (8 years, 2009-2017). For example, adjuvant or salvage epidermal growth factor receptor tyrosine kinase inhibitors were introduced, which are more effective than chemotherapy for patients with *EGFR*-mutant NSCLC.^25^ This may mask the effects of PORT, but it was not considered when the trial was initially designed. Further studies exploring which patients will optimally benefit from PORT are required.

## Conclusions

In this randomized clinical trial of patients with pIIIA-N2 NSCLC after complete resection and adjuvant chemotherapy, PORT did not improve DFS, while it did improve LRFS. Further studies are needed to accurately identify the appropriate patients who will optimally benefit from PORT.

## Notes

Supplement 1. Trial Protocol

Supplement 2. **eAppendix 1.** Diagram **eAppendix 2.** RT parameters

Supplement 3. Data Sharing Statement
