# Supplementary material for: Effect of Postoperative Radiotherapy for Patients With pIIIA-N2 Non–Small Cell Lung Cancer After Complete Resection and Adjuvant Chemotherapy: The Phase 3 PORT-C Randomized Clinical Trial
Source: JAMA Oncol. 2021 Jun 24;7(8):1–8. doi: 10.1001/jamaoncol.2021.1910 (PMC8227450; doi:10.1001/jamaoncol.2021.1910)
Supplement: Supplement 1. — Trial Protocol [file jamaoncol-e211910-s001.pdf]

Postoperative Radiotherapy for Patients with pIIIA-N2 Non-Small Cell Lung Cancer After Complete Resection and Adjuvant Chemotherapy: Study Protocol for a Randomized Controlled Trial

I. Background

Postoperative radiotherapy (PORT) has been widely implemented in clinical practice. Since 1995, a number of randomized controlled studies and meta-analyses have demonstrated that PORT could not improve the survival of patients with non-small cell lung cancer (NSCLC) after surgery and increase the mortality risk for patients with early-stage disease. However, the aforementioned randomized controlled studies were conducted in the era of conventional radiotherapy, and the experiment designs and results interpretations of these studies were also widely controversial. The subgroup analysis results showed that PORT could improve the survival for patients with pathological IIIA-N2 (pIIIA-N2) disease. The main reason for PORT not being beneficial of patients with other stages was the thoracic radiation injuries caused by postoperative conventional radiotherapy, leading to the significant increase of non-cancer related deaths.

Image guided 3-dimensional conformal radiotherapy (3D-CRT) or Intensity Modulated Radiation Therapy (IMRT) has been extensively used in the postoperative treatment for lung cancer since 1990s. This radiotherapy technology is of great advantage in accurately evaluating and optimizing the radiation dose of tumor and thoracic organs at risk, so as to effectively increase the dose to the tumor while strengthening the protection to the normal organs such as the heart and lung. An analysis based on the Surveillance, Epidemiology, and End Results (SEER) database showed that the heart disease-related mortality of patients treated after 1988 was dramatically lower than that of the patients treated before 1988, which was attributed to the widespread use of 3D-CRT after

1988. This result indicated that 3D-CRT could reduce the thoracic radiation injuries. The study by Lally BE *et al* using the data of more than 6000 patients after 1988 from SEER showed that, PORT could improve the 5-year overall survival by 7% for patients with pIIIA-N2 NSCLC. Taken together, the reduction of thoracic radiation toxicities with the use of 3D-CRT may translate into the improvement of the survival for patients treated with PORT. Therefore, the role of PORT for patients with pIIIA-N2 NSCLC needs to be re-evaluated in the setting of 3D-CRT technique. The subgroup analysis of the Adjuvant Navelbine International Trialist Association (ANITA) trial and several smaller randomized controlled studies have shown that PORT improved the 5-year survival for patients with pIIIA-N2 NSCLC. Therefore, postoperative 3D-CRT may be beneficial in improving the survival for NSCLC patients with pIIIA-N2 disease, which needs to be verified by more rigorously designed and larger-scale randomized controlled trials.

Multiple large-scale multicenter randomized controlled studies have shown that postoperative chemotherapy with platinum-based two-drug regimen can improve survival for patients with stage IB-IIIA NSCLC. The ANITA trial showed that the 2-year, 5-year, and 7-year survival rates for patients treated with adjuvant vinorelbine plus cisplatin were 68%, 51%, and 45%, respectively (the corresponding rates for patients in the observation group were 63%, 43%, and 37%, respectively); The 5-year survival rates for patients with stage I, stage II and stage IIIA were 62%, 52%, and 42%, respectively (the corresponding rates for patients in the observation group were 63%, 39%, and 26%, respectively). All these results demonstrated that adjuvant chemotherapy significantly improved the 5-year survival for patients with stage II and stage IIIA-N2 NSCLC after complete resection. The National Cancer Institute of Canada Clinical Trials Group JBR.10 trial showed that adjuvant vinorelbine and cisplatin after resection significantly improved the overall survival for stage IB-II NSCLC, and the main beneficiaries were stage II patients. The International Adjuvant Lung Cancer Trial (IALT) including 1867 patients with stage I-III resected NSCLC demonstrated that, the 5-year overall survival rate and

disease-free survival rate for patients treated with adjuvant chemotherapy including cisplatin and either vinblastine or etoposide were 44.5% and 39.4% (the corresponding rates for patients in the observation group were 40.4% and 34.3%). The 5-year absolute survival benefit was 4.1% and the main beneficiaries were patients with IIIA-N2 disease. Based on the aforementioned studies, postoperative chemotherapy with platinum-based two-drug regimen has been the standard treatment for completed resected NSCLC with stage IB-IIIA disease. In these studies, the local recurrence rate after postoperative chemotherapy for IIIA-N2 patients was still higher than that of stage IB-II, and the subgroup analysis of ANITA trial confirmed that postoperative 3D-CRT could further reduce the local recurrence rate and improve survival for pIIIA-N2 patients. Therefore, it is highly indicated that postoperative 3D-CRT had a beneficial effect on tumor local control and overall survival for pIIIA-N2 NSCLC patients treated with complete resection and adjuvant chemotherapy, but this has not been confirmed by rigorously designed randomized controlled trials.

In addition, NSCLC with pN2 has been identified as a heterogeneous disease including multiple subgroups. According to the position of the involved mediastinal lymph nodes, pN2 disease can be divided into single-station N2 disease with N1 disease, single-station N2 disease without N1 disease (skip metastasis) and multiple-station N2 disease. The first two subgroups account for 20%-30% of patients with pN2 disease after resection, and the prognosis is significantly better than that of the latter subtype. Based on the number or maximum diameter of the metastatic lymph nodes (LNM), pN2 disease can be sub-classified into subgroups of LNM number ≤3 and LNM number >3 or LNM diameter ≤2cm and LNM diameter >2cm, with the prognosis of the former subgroups being better than that of the latter ones. The pN2 disease can also be grouped according to the diagnosis time of N2 disease, and the prognosis of N2 disease diagnosed postoperatively is better than which diagnosed preoperatively. PORT may play different roles in different subgroups of pIIIA-N2 NSCLC patients. The results of a randomized controlled study have already demonstrated that the 5-year survival for patients with

multiple-station N2 disease treated with PORT in addition to adjuvant chemotherapy was 39%, compared with <10% for those who only received adjuvant chemotherapy. Therefore, it is of great significance to investigate the value of PORT in the treatment of different subtypes of pIIIA-N2 NSCLC patients, especially in the modern era with 3D-CRT technique.

In summary, 3D-CRT/IMRT after platinum-based adjuvant chemotherapy can improve the local control rate and survival rate of stage IIIA (N2) non-small cell lung cancer after complete resection, but there is a lack of multicenter randomized controlled trials. At present, ECOG has initiated a multicenter randomized controlled trial of 3D-CRT after adjuvant chemotherapy for stage IIIA (N2) non-small cell lung cancer after complete resection. To determine the value of postoperative adjuvant radiotherapy for stage IIIA (N2) non-small cell lung cancer after complete resection, more parallel multicenter randomized controlled trials of other regions and populations are needed. This study is a single institutional randomized controlled trial of 3D-CRT/IMRT after platinum-based adjuvant chemotherapy of stage IIIA (N2) non-small cell lung cancer with complete resection, which is of great significance to determine the role of postoperative 3D-CRT/IMRT in the multimodality treatment of stage IIIA (N2) non-small cell lung cancer and to improve the standardization level of postoperative 3D-CRT/IMRT.

II. Objectives

1. Primary objective: To evaluate the 3-year disease-free survival (DFS) rate of stage IIIA (N2) NSCLC after complete resection and platinum-based chemotherapy with or without PORT.

2. Secondary objective:

・ Analyze the 3-year overall survival (OS), local-regional free survival (LRFS) and distant metastasis free survival (DMFS) of stage IIIA (N2) NSCLC after complete resection and platinum-based chemotherapy with or without PORT.

・ Analyze the failure pattern of stage IIIA (N2) NSCLC after complete resection and platinum-based chemotherapy with or without PORT.

Analyze the toxicities of stage IIIA (N2) NSCLC after complete

resection and platinum-based chemotherapy with PORT.

III. Methods

1. Study design

This is a phase III randomized controlled clinical trial of platinum-based two-drug chemotherapy and PORT for completely resected stage IIIA (N2) NSCLC.

Stratification factors: number of positive mediastinal lymph nodes (1-3, ＞3), number of detected lymph nodes (≤20, >20).

Randomization: single chemotherapy group (C) - observation group: postoperative platinum-based two-drug chemotherapy for 4 cycles and observation; PORT group - postoperative platinum-based two-drug chemotherapy for 4 cycles, then PORT of 50Gy.

An online system is used to randomize enrolled patients.

2. Number of cases required for the study

Based on the data from our retrospective case-control study, this study is designed to detect an improvement in 3-year DFS from 30% to 44% (equivalent to HR=0.69) at 1-sided type 1 error of 0.025 with 80% power. Assuming a monthly accrual rate of 4.5 patients and guarding against 10% ineligibility or loss to follow-up, the target accrual is 390 patients and the primary analysis is to be performed when at least 230 DFS events were observed.

3. Patient selection

Patients with completely resected NSCLC, who are diagnosed as stage IIIA (N2) by pathology and/or cytology, and without local recurrence or hematogenous metastasis after 4 cycles of platinum-containing two-drug chemotherapy are eligible to be further evaluated. Reasons for choosing such patients:

· Completely resected stage IIIA (N2) NSCLC benefited the most from postoperative adjuvant chemotherapy with platinum-containing two-drug regimen which had become the standard adjuvant therapy for stage IIIA (N2) NSCLC.

· According to the preliminary results of existing randomized controlled clinical trials and the analysis of PORT database based on

multi-centers, the survival rate of stage IIIA (N2) NSCLC after complete resection can be improved by radiotherapy, with the range of 5-15%. However, the role and significance of PORT after postoperative chemotherapy, especially 3D-CRT/IMRT, still need to be confirmed by randomized controlled trials.

- No ethical violation.

3.1 Selection procedure

- Patients who meet the inclusion criteria sign the informed consent after learning details of the trial.
- Patients are formally enrolled after completing and passing all pre-enrollment examinations.

3.2 Inclusion criteria

- Be able to understand the basic information of this study and sign informed consent
- Age 18-70 years old, Eastern Cooperative Oncology Group (ECOG) status (PS)< 2, estimated survival ≥12 months
- Less than 10% weight loss before surgery
- Complete preoperative imaging staging; Accurate description and pathological confirmation of primary lesion and mediastinal lymph node involvement in each station
- The operation was complete resection (lobectomy, sleeve resection); Mediastinal lymph node dissection should at least include: right lung- station 10R/ 7/ 4R, left lung- station 10L/ 7/ 5/ 6/ 4L; R0 resection; Non-small cell lung cancer (NSCLC) with a clear pathologic and/or cytological diagnosis of stage IIIA (N2), including adenocarcinoma (including bronchioloalveolar carcinoma), squamous cell carcinoma, large cell carcinoma, or mixed (squamous cell carcinoma and adenocarcinoma)
- Postoperative lung function examination: FEV_1 > 1 L (or greater than 35% expected value, PO2 ≥ 70 mm Hg, PCO2 < 45 mm Hg)
- Four cycles of postoperative chemotherapy with platinum -containing two-drug regimen

- Radiotherapy is planned to start at least 2 weeks after adjuvant chemotherapy, all examinations excluded local recurrence and hematogenous metastasis
- No serious medical disease and major organ dysfunction; Blood routine, hepatic, renal and cardiac function normal
- Good compliance and easy to follow up

3.3 Exclusion criteria

- Postoperative lung function examination: FEV_1≤1 L (or less than 35% expected value, PO2 < 70 mm Hg, PCO2 ≥ 45 mm Hg)
- Greater than 10% weight loss before surgery
- Pneumonectomy
- Preoperative neoadjuvant chemotherapy, targeted therapy at any time prior to radiotherapy or enrollment
- Recurrence or metastasis occurred
- More than eight weeks after completion of adjuvant chemotherapy
- Serious medical problems and major organ dysfunction
- Hepatic and renal insufficiency
- history of serious or uncontrolled heart disease
- Uncontrolled diabetes or high blood pressure
- Active gastrointestinal ulcer
- History of mental illness
- severe drug allergies
- Pregnancy, lactation, or nullipara
- Unable to understand and express informed consent
- Researchers considered inappropriate to participate in this study

3.4 Rejection Criteria:

- Violate the inclusion criteria:
- Not following the treatment plan
- Patients who withdraw from the study due to adverse events wouldn’t evaluate the efficacy, but side effects.
- The main indicators or important clinical data are incomplete

3.5 Exit Criteria:

· Any recurrence during the treatment

· Patients request to withdraw from the study

· Non-hematologic toxicity above grade 3 (CTC AEs 3.0) (excluding nausea, vomiting, hair loss), failure to recover after symptomatic treatment, or serious adverse events

· Researchers believe that it is appropriate to withdraw from the study

· Anyone who delays treatment for more than 2 weeks (beyond the treatment or inspection time specified in the schedule)

4. Study Design

4.1 Treatment plan design

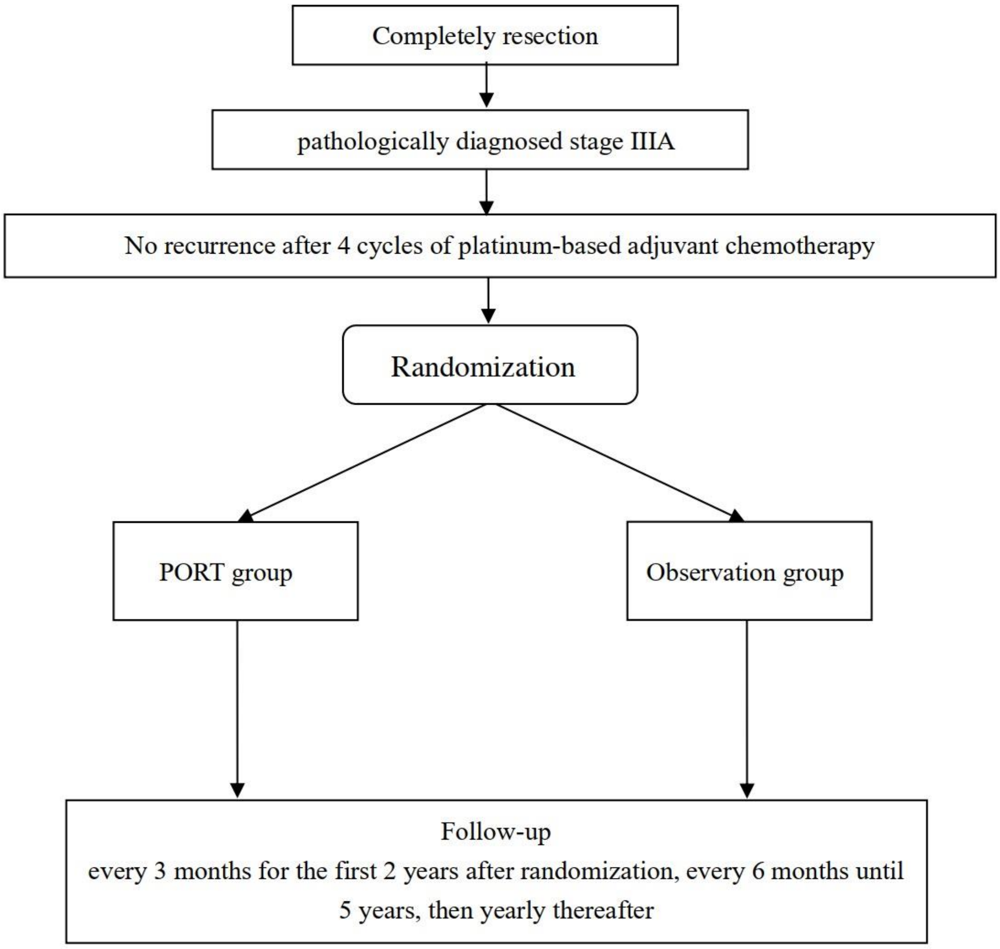

4.2 Radiotherapy plan

4.2.1 Body position fixation and CT simulation

This clinical trial should limit breathing as much as possible to improve

target dose and protect normal tissue. It is recommended to perform CT simulation under the condition of calmness and body position fixation by mesh cover to restrict breath. It is recommended to use enhanced CT scan. The scan layer thickness is 0.5cm and the scan range is from the cricothyroid membrane to the inferior of the first lumbar vertebral, including the supraclavicular, lung, mediastinum, upper abdomen and adrenal gland.

4.2.2 Definition of radiotherapy target volume

The window width and window level should be fixed when the image workstation defines the target volume. The lung window, window width is 1600 and window level is -600.The vertical window, window width is 400 and window level is 20. Before the target volume is defined, the mobility of the mediastinum in three dimensional the front and rear, up and down (head and foot) and left and right directions is determined under fluoroscopy or 4DCT.

When the target volume is defined, the mediastinal lymph node regions of each station follows the definition of AJCC, referring to Michgan University's map outline and boundary. Target volume definition principle: The right lung PORT CTV includes right hilar, subcarinal, mediastinal lymph nodes (4R, 2R); left lung PORT CTV includes left hilar, subcarinal, mediastinum and lymph node station (4L, 5, 6, 2L). Stumps of central lesions should also be included in CTV.

Based on the definition of CTV, the measured mobility and placement error are added to form a planning target volume (PTV).

4.2.3 Radiotherapy plan evaluation

After the completion of 3D-CRT/IMRT plan, target dose and organs dose at risk should be evaluated. Dose volume histogram (DVH) is used as the basic tool to evaluate the dose of PTV distribution and organs at risk according to the distribution of regional isodose curve in three-dimensional space.

Dose distribution requirements: in consideration of dose uniformity and cold spot/hot spot distribution, 95% of the planned irradiation volume (PTV) is required to receive prescription dose irradiation, with dose uniformity of 95-107%. If necessary, the maximum dose limit in the target area can be

appropriately liberalized, but the maximum dose should not exceed 10% of the above prescription dose.

Dose limitations for organs at risk: all lung V20 < 25% , mean lung dose < 12Gy , maximum dose of spinal cord less than 45 Gy; heart V30 < 40% , heart V40 < 30%, pay attention to avoiding the dose of left ventricle more than 40Gy; V60 < 50% for esophagus; V30 < 40% for liver.

Total dose and fractionation schemes: 2Gy / F conventional fractionation radiotherapy, total dose 50Gy / 25F / 5W.

4.2.4 Radiotherapy implementation and quality assurance

The definition of treatment target area should be drawn and checked by at least two radiation oncologists. All patients should be adopted to the same imaging restraint conditions including enhancement delay time, window width and window position.

Before radiotherapy, the treatment position and dosimetry should be verified according to the working standards.

EPID imaging verification should be carried out at the beginning and during the treatment, and offline correction should be carried out if necessary. If image guided radiotherapy equipment such as IGRT is available, online and real-time correction of IGRT should be carried out.

Target definition images, EPID verification images or IGRT images, and radiotherapy records of all enrolled patients should be kept for at least 5 years for future reference.

4.2.5 Treatment of toxicities caused by PORT

Antibiotics can be used when patients suffer ≥ grade 2 radiation trachea reaction and radiation pneumonia. Corticosteroids can be used if necessary, and narcotic antitussive drugs can be used in severe cough.

The patients with ≥ grade 2 radiation esophagitis can be treated with antibiotics for a short time, and those with severe pain can be treated with narcotic analgesics to relieve symptoms.

If treatments for any ≥ grade 3 non-hematologic toxic treatment could not be relieved, the radiotherapy should be suspended. If the researchers consider that the patients are no longer suitable for further treatment or they ask for dropping out for any reason, the patients should be quit.

4.3. Time of radiotherapy

The time of the first radiotherapy should be no later than 6 weeks after the end of adjuvant chemotherapy. During the treatment, the total interruption of treatment for any factor could not exceed 10 days.

4.4 Concomitant medication

During the clinical study, other drugs related to tumor treatment should be stopped. When severe toxicities such as radiation esophagitis, skin injury, radiation pneumonia and other symptoms happen, drugs for symptomatic treatment can be used.

4.5 Basic inspection requirements

4.5.1 The following staging by the conventional examinations should be completed before operation:

Chest CT or MRI, brain MRI, bone scan (if positive, bone metastasis must be confirmed by MRI or CT of corresponding parts, otherwise bone metastasis should not be diagnosed). Abdominal organ and lymph node metastasis can be excluded by B-ultrasound. Complete accurate preoperative staging.

4.5.2 The following examinations should be completed before radiotherapy:

Collect medical history: fill in the clinical medical records according to the requirements of CRF form;

ECOG;

Vital signs, including heart rate, respiration, blood pressure, body temperature, etc;

Blood, urine and stool routine, blood sugar, blood biochemical examination including liver function, renal function and electrolyte, electrocardiogram;

Relevant imaging examination: one must be done for chest CT or MRI to exclude local or mediastinal lymph node recurrence; brain MRI is used to exclude brain metastasis; if bone scan indicates positive, bone metastasis must be confirmed by MRI or CT of corresponding parts, otherwise bone metastasis cannot be diagnosed; abdominal organ and lymph node metastasis can be excluded by B-ultrasound.

Special examination of tumor markers: CEA, cryfra 21-1, CA125, NSE, etc.

4.5.3 Examination during the study

Vital signs and physical examination: once a week;

Blood routine test: regular examination according to chemotherapy, usually once a week;

Urine, stool routine and blood sugar, liver function, renal function and electrolyte examination: before and after radiotherapy;

Imaging examination: when the patients finish the treatment and during the follow-up. The methods of imaging examination should be the same as recruitment;

Tumor markers: when the pts finish the treatment and when they follow-up.

4.6 Follow-up

All subjects will be followed up. Starting and ending time: From the date of pathological diagnosis to the date when the patient dies. Frequency of follow-up: every 3 months for the first 2 years after randomization, every 6 months until 5 years, then yearly thereafter.

5. Clinical evaluation

The primary endpoint of this study is to evaluate the 3-year DFS, as well as OS, LRFS, DMFS, the pattern of failures and toxicities of complete resection of stage IIIA (N2) NSCLC after chemotherapy with or without PORT.

5.1 Definitions

5.1.1 DFS

DFS is defined as the duration between randomization to any disease recurrence or death due to any cause.

5.1.2 OS

OS is defined as the duration between randomization to death due to any cause.

5.1.3 LRFS

LRFS is defined as the duration between randomization to local-regional recurrence or death due to any cause.

5.1.4 DMFS

DMFS is defined as the duration between randomization to distant metastasis or death due to any cause.

5.1.5 Criteria for disease recurrence.

Any of the following conditions happened at any time during follow-up is regarded as recurrence.

Measurable lesions are those can be measured by clinical or imaging methods. The double diameter of intrapulmonary lesion measured in X-ray chest film ≥ 10 mm × 10 mm, in ordinary CT or MRI scan ≥ 20 mm × 20 mm and in spiral CT scan diameter ≥ 10 mm × 10 mm.

The evaluable lesions: single diameter measurable lesions, obscure boundary masses, small lesions that unable to be measured (such as miliary or patchy lesions in the lung), lesions with both diameters less than 10 mm, masses with diameters less than scan spacing, and so on.

Unevaluable lesions: the osteogenic metastasis, pleural effusion, ascites, pericardial effusion, intrapulmonary carcinomatous lymphangitis and patients with lesions that had been treated with radiotherapy in the past and had no progression.

Examination methods: CT or MRI, B-mode ultrasound, X-ray, ECT, PET and body surface photography, it can be selected according to the condition of the disease. Time of examination: within 1 week before radiotherapy, within 3 days after radiotherapy and during follow-up.

5.2 Safety evaluation

5.2.1 Adverse events

5.2.1.1 Definition

Any adverse medical event that occurs from the time the patient signed the informed consent and enrolled in the trial to the last follow-up, regardless of whether it has a causal relationship with the trial or not, is considered an adverse event.

Adverse events should be recorded in detail during the trial, including the time of occurrence, severity, duration, treatments, and outcomes.

5.2.1.2 Evaluation criteria for adverse events:

The toxicities are evaluated according to the CTC AEs 3.0.

5.2.1.3 Evaluation criteria for the relationship between adverse events and the trial

The investigators should evaluate the possible relationship between adverse events and the trial, with reference to the following criteria:

Definitely relevant: The occurrence time and type of adverse event are consistent with that of radiation damage.

Probably relevant: The occurrence time and type of adverse event are consistent with that of radiation damage, but it may also be probably caused by the patient's clinical status or other treatment modalities.

Probably irrelevant: The occurrence time and type of adverse event are not well consistent with that of radiation damage, and it may also be probably caused by the patient's clinical status or other treatment modalities.

Irrelevant: The occurrence time and type of adverse event are not consistent with that of radiation damage and it may also be probably caused by the patient's clinical status or other treatments. If the patient's clinical status is improved or other treatments are stopped, the event would disappear. If other treatment were conducted again, it would appear as well.

Uncertainty: The occurrence time and type of adverse event are not clearly consistent with that of radiation damage. It may also be probably caused by other drugs.

5.2.2 Serious adverse events

5.2.2.1 Definition of serious adverse events

- Death
- Life-threatening
- Hospitalization or extended hospitalization time
- Permanent or severely disabled
- Congenital malformations or defects

5.2.2.2 Reporting system

Any serious adverse reactions that occur during or within 30 days of the last treatment of the trial, regardless of whether it relates to this trial or not, should be reported to the principal investigator (PI), the principal of the

clinical sponsor and the ethics committee by telephone within 24 hours, and should be reported to the State Drug Administration (SDA) by the sponsor.

6. Ethics

6.1 Informed consent

Physicians should provide a complete and comprehensive overview of the study's purpose, possible benefits, possible adverse effects, and related risks, to the patients or their designated representatives. Patients should be informed their rights, benefits, and risks. The informed consent should be signed before enrollment and filed in the case report form (CRF).

6.2 Ethics and policies

This clinical trial follows the Helsinki Declaration (1996 edition), the Good Clinical Practice (GCP) issued by SDA and related regulations. The trial must be approved by the ethics committee before the implementation. Any changes to the program during the clinical trial should be reported to the ethics committee and filed.

7. Quality assurance.

To ensure that this trial can be carried out strictly according to the clinical research protocol, the clinical researchers and sponsor should operate it in strict accordance with the requirements of the Good Clinical Practice (GCP) standard strictly during the whole process of the trial, making sure that the procedure is standardized, the data is accurate and the conclusion is reliable. Specific requirements are as follows:

7.1 Requirements for the sponsors

· Provide Investigator's Brochure and related literature for researchers. Give lectures on clinical design explanation and CRF filling to researchers before the initiation of the trail.

· Dispatch clinical research associates to perform on-site supervision.

· Guarantee to keep in touch with researchers by phone, fax or email.

· Provide online service for randomized enrollment.

7.2 Requirements for researchers

- Collect informed consent signed by each subject or his/her agent.
- Carefully fill in the Case Report Form (CRF) as required.
- Provide good cooperation with the regular visits of clinical research associates appointed by the sponsor.
- Fully preserve the laboratory examination records, clinical records and original medical records of patients.

8. Data processing and preservation

8.1 Case Report Form (CRF)

The CRFs should be filled in daily by the investigators to ensure the accuracy of contents and the timeliness of summary. The CRFs should not be altered generally. If there is any error which has to be modified, the modification should be signed (read in instructions of the CRF filling). All CRFs are kept in our institution. The completed CRFs are verified by the clinical research associates and then the data entry is conducted. The content of the CRFs cannot be modified henceforth.

8.2 Database establishment

After receiving the CRFs, the statisticians will forward any questions to the researchers through clinical research associates for verification. The researchers should respond and return as soon as possible. Then statisticians should establish the database in time. After the database is verified, the data will be locked up by the main researcher, sponsor, statistician and clinical research associate to ensure the data security. And all data must be backed up.

8.3 Statistics

8.3.1 Sample size

Based on the data from our retrospective case-control study, this study is designed to detect an improvement in 3-year DFS from 30% to 44% (equivalent to HR=0.69) at 1-sided type 1 error of 0.025 with 80% power. Assuming a monthly accrual rate of 4.5 patients and guarding against 10% ineligibility or loss to follow-up, the target accrual is 390 patients and the primary analysis is to be performed when at least 230 DFS events were observed.

8.3.2 Statistical analysis

All statistical tests are performed and 2-sided P≤0.05 is considered statistically significant (primary endpoint at 1-sided 0.025 per study design). The quantitative indicators contain mean, standard deviation, median, minimum and maximum number. The qualitative indicators contain the number and percentage of each variable. The data of each group are calculated respectively, and Wilcoxon or log rank test is used to compare and analyze the survival data of the two groups.

8.4 Preservation of data

The researchers should preserve all relevant data intactly. And the data should be kept for more than 5 years by researchers according to China's GCP principle.

10. Clinical study sponsor: Department of Radiation Oncology, National Cancer Center/National Clinical Research Center for Cancer/Cancer Hospital, Chinese Academy of Medical Sciences and Peking Union Medical College, Beijing, China.

```
require(survival)

require(pec)

require(rms)

library(Publish)

library(survival)

library(data.table)

require(ggpubr)

require(survminer)
```

```
port <- read.csv('./dataset/RCT_data_forR.csv')

port$trt0 <- 1*(port$trt=='PORT') # cmprsk need a numeric treatment indicator

port$cN2 <- 1*(port$cN2.groups==1)



setDT(port)

port[,':='(gender=factor(gender, levels=c(1,2), labels=c('Male','Female')),

           age60=factor(age60,levels=c(1,2), labels=c('Age <=60 years','Age >60 years')),

           smoking=factor(smoking, level=c(0,1), labels=c('No Smoking','Smoking')),

           KPS.groups=factor(KPS.groups, levels=c(1,2),labels = c('KPS >=90','KPS <90')),

           tumor.location=factor(tumor.location,levels=c(1,2), labels=c('Left','Right')),

           inv.visceral=factor(inv.visceral, levels=c(0,1), labels=c('No','Yes')),

           path.scc=factor(path.scc, levels=c(0,1), labels=c('NonSquamous','Squamous')),

           postop_dmax=factor(postop_dmax, levels=c(1,2),

                              labels=c('postop dmax<=3cm','postop dmax>3cm')),

           pT.groups2=factor(pT.groups2, levels=c(1,2),

                             labels = c('Path T1','Path T2-T3')),

           detected.LN.groups=factor(detected.LN.groups, levels = c(1,2),
```

```
                                labels = c('Lymph Nodes <=20','Lymph Nodes > 20')),

          LNM.groups3=factor(LNM.groups3, levels=c(1,2),

                           labels = c('Pos Lymph Nodes <= 3','Pos Lymph Nodes >3')),

          EGFR = factor(EGFR, levels = c(0,1),

                       labels = c('EGFR Sensitive','EGFR non-Sensitive')),

          cN2 = factor(cN2, levels=c(0,1), labels=c('Not cN2', 'cN2'))

)]



table(port$trt); table(port$gender); table(port$age60); table(port$smoking); table(port$KPS.groups)

table(port$tumor.location); table(port$inv.visceral);

table(port$path.scc); table(port$postop_dmax); table(port$pT.groups2); table(port$detected.LN.groups);

table(port$LNM.groups3); table(port$EGFR); table(port$cN2.groups)



#### Data Prep for competing risk event indicator

port$etype <- 1*((port$LRFS_status==1)&(port$LRFS_months<port$OS_months)&(round(port$LRFS_months,4)==round(port$DFS_months,4))) +


2*((port$DMFS_status==1)&(port$DMFS_months<port$OS_months)&(round(port$DMFS_months,4)==round(port$DFS_months,4))) +


4*((port$DFS_status==1)&(port$OS_status==1)&(round(port$DFS_months,4)==round(port$OS_months,4)))
```

```
table(port$etype)

port$etype <- factor(port$etype)

# etype = 1,  LR only

# etype = 2,  DM only

# etype = 3,  DM+LR

# etype = 4   Death without Progression


### per-protocol indicator

port$pp       <-       1*(port$port_rcv==1)*(port$trt=="PORT")       +
1*(port$port_rcv==2)*(port$trt=="Obs")

table(port$pp)


### as-treated indicator

port$at <- 1*(port$port_rcv==1)

table(port$at)


#### Median follow-up

fu <- survfit(Surv(OS_months,OS_status==0) ~ 1 , data=port)

quantile(fu)


################      DFS
```

```
cox.dfs.itt <- coxph(Surv(DFS_months,DFS_status) ~ trt , data=port)

summary(cox.dfs.itt)

## logrank for DFS, and 1-sided p-value

lgrk.dfs <- survdiff(Surv(DFS_months,DFS_status) ~ trt, data=port)

(dfs.itt.pval <- pchisq(lgrk.dfs$chisq, df=1, lower.tail = F)/2)


# unadjusted KM

km.dfs <- survfit(Surv(DFS_months,DFS_status) ~ trt , data=port)


###################### OS

cox.os.itt <- coxph(Surv(OS_months,OS_status) ~ trt , data=port)

summary(cox.os.itt)


lgrk.os <- survdiff(Surv(OS_months,OS_status) ~ trt, data=port)

(os.itt.pval <- pchisq(lgrk.os$chisq, df=1, lower.tail = F))


# unadjusted KM

km.os <- survfit(Surv(OS_months,OS_status) ~ trt , data=port)


###################### LRFS

cox.lrfs.itt <- coxph(Surv(LRFS_months,LRFS_status) ~ trt , data=port)

summary(cox.lrfs.itt)
```

```
lgrk.lrfs <- survdiff(Surv(LRFS_months,LRFS_status) ~ trt, data=port)
(lrfs.itt.pval <- pchisq(lgrk.lrfs$chisq, df=1, lower.tail = F))

# unadjusted KM
km.lrfs <- survfit(Surv(LRFS_months,LRFS_status) ~ trt , data=port)

###################### DMFS
cox.dmfs.itt <- coxph(Surv(DMFS_months,DMFS_status) ~ trt , data=port)
summary(cox.dmfs.itt)

lgrk.dmfs <- survdiff(Surv(DMFS_months,DMFS_status) ~ trt, data=port)
(dmfs.itt.pval <- pchisq(lgrk.dmfs$chisq, df=1, lower.tail = F))

# unadjusted KM
km.dmfs <- survfit(Surv(DMFS_months,DMFS_status) ~ trt , data=port)


############## PP analysis
port.pp <- subset(port, pp==1)
#### DFS
cox.dfs.pp <- coxph(Surv(DFS_months,DFS_status) ~ trt , data=port.pp)
```

```
summary(cox.dfs.pp)


## logrank for DFS, and 2-sided p-value

lgrk.dfs.pp <- survdiff(Surv(DFS_months,DFS_status) ~ trt, data=port.pp)

(dfs.pp.pval <- pchisq(lgrk.dfs.pp$chisq, df=1, lower.tail = F))


############## OS

cox.os.pp <- coxph(Surv(OS_months,OS_status) ~ trt , data=port.pp)

summary(cox.os.pp)


lgrk.os.pp <- survdiff(Surv(OS_months,OS_status) ~ trt, data=port.pp)

(os.pp.pval <- pchisq(lgrk.os.pp$chisq, df=1, lower.tail = F))



###################### LRFS

cox.lrfs.itt <- coxph(Surv(LRFS_months,LRFS_status) ~ trt , data=port.pp)

summary(cox.lrfs.itt)


lgrk.lrfs <- survdiff(Surv(LRFS_months,LRFS_status) ~ trt, data=port.pp)

(lrfs.itt.pval <- pchisq(lgrk.lrfs$chisq, df=1, lower.tail = F))


# unadjusted KM
```

```
km.lrfs <- survfit(Surv(LRFS_months,LRFS_status) ~ trt , data=port.pp)


###################### DMFS
cox.dmfs.itt <- coxph(Surv(DMFS_months,DMFS_status) ~ trt , data=port.pp)
summary(cox.dmfs.itt)


lgrk.dmfs <- survdiff(Surv(DMFS_months,DMFS_status) ~ trt, data=port.pp)
(dmfs.itt.pval <- pchisq(lgrk.dmfs$chisq, df=1, lower.tail = F))


# unadjusted KM
km.dmfs <- survfit(Surv(DMFS_months,DMFS_status) ~ trt , data=port.pp)


###################### AT analysis
## DFS Univariate Cox
cox.dfs.at <- coxph(Surv(DFS_months,DFS_status) ~ at , data=port)
summary(cox.dfs.at)


## logrank for DFS, and 2-sided p-value
lgrk.dfs.at <- survdiff(Surv(DFS_months,DFS_status) ~ at, data=port)
(dfs.at.pval <- pchisq(lgrk.dfs.at$chisq, df=1, lower.tail = F))


############## OS
```

```
## Univariate Cox

cox.os.at <- coxph(Surv(OS_months,OS_status) ~ at , data=port)

summary(cox.os.at)


## logrank for DFS, and 2-sided p-value

lgrk.os.at <- survdiff(Surv(OS_months,OS_status) ~ at, data=port)

(os.at.pval <- pchisq(lgrk.os.at$chisq, df=1, lower.tail = F))




###################### LRFS

cox.lrfs.itt <- coxph(Surv(LRFS_months,LRFS_status) ~ at , data=port)

summary(cox.lrfs.itt)


lgrk.lrfs <- survdiff(Surv(LRFS_months,LRFS_status) ~ at, data=port)

(lrfs.itt.pval <- pchisq(lgrk.lrfs$chisq, df=1, lower.tail = F))


# unadjusted KM

km.lrfs <- survfit(Surv(LRFS_months,LRFS_status) ~ at , data=port)


###################### DMFS

cox.dmfs.itt <- coxph(Surv(DMFS_months,DMFS_status) ~ at , data=port)

summary(cox.dmfs.itt)
```

```
lgrk.dmfs <- survdiff(Surv(DMFS_months,DMFS_status) ~ at, data=port)

(dmfs.itt.pval <- pchisq(lgrk.dmfs$chisq, df=1, lower.tail = F))


# unadjusted KM

km.dmfs <- survfit(Surv(DMFS_months,DMFS_status) ~ at , data=port)




#### Stratified analysis

## DFS

lgrks.dfs <- survdiff(Surv(DFS_months,DFS_status) ~ trt + strata(detected.LN.groups, LNM.groups3), data=port)

(dfss.itt.pval <- pchisq(lgrks.dfs$chisq, df=1, lower.tail = F)/2)


## stratified Cox model

coxs.dfs.itt <- coxph(Surv(DFS_months,DFS_status) ~ trt + strata(detected.LN.groups, LNM.groups3), data=port)

summary(coxs.dfs.itt)


## OS

lgrks.os <- survdiff(Surv(OS_months,OS_status) ~ trt + strata(detected.LN.groups, LNM.groups3), data=port)

(oss.itt.pval <- pchisq(lgrks.os$chisq, df=1, lower.tail = F))
```

```
## stratified Cox model

coxs.os.itt <- coxph(Surv(OS_months,OS_status) ~ trt + strata(detected.LN.groups, LNM.groups3), data=port)

summary(coxs.os.itt)




#### Figure

dfs.itt <- ggsurvplot(km.dfs, data = port,

                      title = "ITT Population",

                      xlab = 'Months',

                      ylab = 'Disease-free Survival',

                      pval = paste0('2-sided log-rank p',ifelse(dfs.itt.pval<0.001,"<.001",paste0('=',round(dfs.itt.pval,2)) ) ),

                      surv.median.line = "hv",            # Add median survival lines

                      legend.title = "Treatment",                  # Change legend titles

                      legend.labs = c("Observation","PORT"),        # Change legend labels

                      palette = "jama",                    # Use JCO journal color palette

                      risk.table = TRUE,                  # Add No at risk table

                      tables.height = 0.15,               # Specify
```

```
tables height
                      tables.theme = theme_cleantable(),  # Clean theme
for tables
                      tables.y.text = FALSE,              # Hide tables
y axis text
                      xlim = c(0,84),
                      break.x.by = 12,
                      font.x=c(14),
                      font.y=c(14,"bold.italic"),
                      censor.size = 2,
                      ggtheme = theme_classic() # Change ggplot2 theme
)


os.itt <-  ggsurvplot(km.os, data = port,
                      title = "ITT Population",
                      xlab = 'Months',
                      ylab = 'Overall Survival',
                      pval = paste0('2-sided log-rank
p',ifelse(os.itt.pval<0.001,"<.001",paste0('=',round(os.itt.pval,2)))
),
                      surv.median.line = "hv",            # Add median
survival lines
                      legend.title = "Treatment",                     #
Change legend titles
                      legend.labs = c("Observation","PORT"),          #
Change legend labels
```

```
                      palette = "jama",                      # Use JCO
journal color palette
                      risk.table = TRUE,                    # Add No at
risk table
                      tables.height = 0.15,                  # Specify
tables height
                      tables.theme = theme_cleantable(),   # Clean theme
for tables
                      tables.y.text = FALSE,               # Hide tables
y axis text
                      xlim = c(0,84),
                      break.x.by = 12,
                      font.x=c(14),
                      font.y=c(14,"bold.italic"),
                      censor.size = 2,
                      ggtheme = theme_classic() # Change ggplot2 theme
)



dfs.pp <-  ggsurvplot(km.dfs.pp, data = port.pp,
                      title = "Per Protocol Population",
                      xlab = 'Months',
                      ylab = 'Disease-free Survival',
                      pval       =       paste0('2-sided       log-rank
p',ifelse(dfs.pp.pval<0.001,"<.001",paste0('=',round(dfs.pp.pval,2)))
```

```
),
                    surv.median.line = "hv",            # Add median survival lines
                    legend.title = "Treatment",                  # Change legend titles
                    legend.labs = c("Observation","PORT"),        # Change legend labels
                    palette = "jama",                        # Use JCO journal color palette
                    risk.table = TRUE,                   # Add No at risk table
                    cumevents = TRUE,                          # Add cumulative No of events table
                    tables.height = 0.15,                 # Specify tables height
                    tables.theme = theme_cleantable(),  # Clean theme for tables
                    tables.y.text = FALSE,                # Hide tables y axis text
                    xlim = c(0,84),
                    break.x.by = 12,
                    font.x=c(14),
                    font.y=c(14,"bold.italic"),
                    censor.size = 2,
                    ggtheme = theme_classic() # Change ggplot2 theme
)
```

```
os.pp <-  ggsurvplot(km.os.pp, data = port.pp,

                     title = "Per Protocol Population",

                     xlab = 'Months',

                     ylab = 'Overall Survival',

                     pval = paste0('2-sided log-rank p',ifelse(os.pp.pval<0.001,"<.001",paste0('=',round(os.pp.pval,2))) ),

                     surv.median.line = "hv",            # Add median survival lines

                     legend.title = "Treatment",              # Change legend titles

                     legend.labs = c("Observation","PORT"),    # Change legend labels

                     palette = "jama",                      # Use JCO journal color palette

                     risk.table = TRUE,                  # Add No at risk table

                     tables.height = 0.15,                 # Specify tables height

                     tables.theme = theme_cleantable(),  # Clean theme for tables

                     tables.y.text = FALSE,              # Hide tables y axis text

                     xlim = c(0,84),

                     break.x.by = 12,

                     font.x=c(14),

                     font.y=c(14,"bold.italic"),
```

```
                    censor.size = 2,
                    ggtheme = theme_classic() # Change ggplot2 theme
)

dfs.itt.all <- ggarrange(dfs.itt$plot, dfs.itt$table, heights = c(2, 0.5),
                         ncol = 1, nrow = 2, align = "v")

os.itt.all <- ggarrange(os.itt$plot, os.itt$table, heights = c(2, 0.5),
                        ncol = 1, nrow = 2, align = "v")

dfs.pp.all <- ggarrange(dfs.pp$plot, dfs.pp$table, heights = c(2, 0.5),
                        ncol = 1, nrow = 2, align = "v")

os.pp.all <- ggarrange(os.pp$plot, os.pp$table, heights = c(2, 0.5),
                       ncol = 1, nrow = 2, align = "v")

fig2 <- ggarrange(dfs.itt.all, os.itt.all, dfs.pp.all, os.pp.all, nrow=2, ncol=2,
                  labels=c("A","B","C","D"))

ggexport(fig2, filename = './manuscript/Figure 2 - Combined.tiff', width = 800, height = 800)
```

```
require(cmprsk)

require(mstate)


######################### Gray's test

cif.cmprsk <- cuminc(port$DFS_months,port$etype,port$trt)

gray.pval <- cif.cmprsk$Tests


#### CIF plots


jpeg(file="./manuscript/Figure 3 - Failure Pattern.jpg", height=8,width=16, units= "in", pointsize = 1/300, res=300)

#tiff(file="./manuscript/Figure 3 - Failure Pattern.tiff", height=8,width=16, units= "in", pointsize = 1/300, res=300)


par(mfrow=c(1,2))

max.x <- 84


library(RColorBrewer)

coul = brewer.pal(4, "Pastel2")


#### Obs.
```

```
cif <- survfit(Surv(DFS_months,DFS_status) ~ 1, etype=etype, data=port.0)

mtitle <- "Observation"

# plot

cif.1 <- c(0, cif$pstate[,2]); cif.2 <- c(0, cif$pstate[,3]); cif.3 <- c(0, cif$pstate[,4]); cif.4 <- c(0, cif$pstate[,5]);

cif.time <- c(0, cif$time)



line.1 <- cif.4 # death only

line.2 <- cif.4+cif.1 # + LR

line.3 <- cif.4+cif.1+cif.3 # + LR/DM

line.4 <- cif.4+cif.1+cif.3+cif.2 # + DM



plot(cif.time,cif.4,type='s',lwd=3,col=1,xlim=c(0,max.x),ylim=c(0,1),

     cex.lab=1.5,cex.axis=1.3, cex.main=2, axes=F,

     xlab='Months from Randomization',ylab='Cumulative Incidence')

axis(1, at = seq(0,max.x,by=12),cex.axis=1.5)

axis(2, at = seq(0,1.1,by=0.1),cex.axis=1.1)

title(mtitle,cex=2)



lines(cif.time,line.2,type='s',lwd=3,col=2)

lines(cif.time,line.3,type='s',lwd=3,col=3)

lines(cif.time,line.4,type='s',lwd=3,col=4)
```

```
polygon(rep(cif.time,each=2),
c(0,rep(line.1[-length(line.1)],each=2),0),

        col='gray')


polygon(c(rep(cif.time,each=2),rev(rep(cif.time,each=2))),

c(0,rep(line.2[-length(line.2)],each=2),0,rev(c(0,rep(line.1[-length(line.1)],each=2),0))),

        #density=c(2, 10), angle=c(45, -45),col=2)

        col=coul[1])


polygon(c(rep(cif.time,each=2),rev(rep(cif.time,each=2))),

c(0,rep(line.3[-length(line.3)],each=2),0,rev(c(0,rep(line.2[-length(line.2)],each=2),0))),

        #density=c(3, 10), angle=c(90, -45),col=3)

        col=coul[2])


polygon(c(rep(cif.time,each=2),rev(rep(cif.time,each=2))),

c(0,rep(line.4[-length(line.4)],each=2),0,rev(c(0,rep(line.3[-length(line.3)],each=2),0))),

        #density=c(4, 10), angle=c(135, -45),col=4)

        col=coul[3])
```

```
polygon(rep(cif.time,each=2),
c(1,rep(line.4[-length(line.4)],each=2),1),

        #density=c(2, 10),  angle=c(0, -45),col=6)

        col=coul[4])



text(max.x-25,-0.02,'Death without Progression',cex=1.5)

text(max.x-10,0.05,'LR only',cex=1.5)

text(max.x-10,0.25,'LR+DM',cex=1.5)

text(max.x-10,0.6,'DM only',cex=1.5)

text(max.x-10,0.9,'Disease-Free',cex=1.5)



# save CIF for Obs.

cif.0 <- cif; cif.0.lr <- cif.1; cif.0.dm <- cif.2; cif.0.lrdm <- cif.3;
cif.0.d <- cif.4



#### PORT

cif <- survfit(Surv(DFS_months,DFS_status) ~ 1, etype=etype, data=port.1)

mtitle <- "PORT"

# plot

cif.1 <- c(0, cif$pstate[,2]); cif.2 <- c(0, cif$pstate[,3]); cif.3 <- c(0,
cif$pstate[,4]); cif.4 <- c(0, cif$pstate[,5]);

cif.time <- c(0, cif$time)
```

```
line.1 <- cif.4 # death only

line.2 <- cif.4+cif.1 # + LR

line.3 <- cif.4+cif.1+cif.3 # + LR/DM

line.4 <- cif.4+cif.1+cif.3+cif.2 # + DM


plot(cif.time,cif.4,type='s',lwd=3,col=1,xlim=c(0,max.x),ylim=c(0,1),

     cex.lab=1.5,cex.axis=1.3, cex.main=2, axes=F,

     xlab='Months from Randomization',ylab='Cumulative Incidence')

axis(1, at = seq(0,max.x,by=12),cex.axis=1.5)

axis(2, at = seq(0,1.1,by=0.1),cex.axis=1.1)

title(mtitle,cex=2)


lines(cif.time,line.2,type='s',lwd=3,col=2)

lines(cif.time,line.3,type='s',lwd=3,col=3)

lines(cif.time,line.4,type='s',lwd=3,col=4)


polygon(rep(cif.time,each=2),
c(0,rep(line.1[-length(line.1)],each=2),0),

        col='gray')


polygon(c(rep(cif.time,each=2),rev(rep(cif.time,each=2))),


c(0,rep(line.2[-length(line.2)],each=2),0,rev(c(0,rep(line.1[-length(l
ine.1)],each=2),0))),
```

```
        #density=c(2, 10), angle=c(45, -45),col=2)

        col=coul[1])


polygon(c(rep(cif.time,each=2),rev(rep(cif.time,each=2))),


c(0,rep(line.3[-length(line.3)],each=2),0,rev(c(0,rep(line.2[-length(line.2)],each=2),0))),

        #density=c(3, 10), angle=c(90, -45),col=3)

        col=coul[2])


polygon(c(rep(cif.time,each=2),rev(rep(cif.time,each=2))),


c(0,rep(line.4[-length(line.4)],each=2),0,rev(c(0,rep(line.3[-length(line.3)],each=2),0))),

        #density=c(4, 10), angle=c(135, -45),col=4)

        col=coul[3])


polygon(rep(cif.time,each=2),
c(1,rep(line.4[-length(line.4)],each=2),1),

        #density=c(2, 10),  angle=c(0, -45),col=6)

        col=coul[4])


text(max.x-25,-0.02,paste0('Death without Progression, p=',round(gray.pval[4,2],3)),cex=1.5)

text(max.x-10,0.05,paste0('LR only,
```

```
p=',round(gray.pval[1,2],3)),cex=1.5)

text(max.x-10,0.20,paste0('LR+DM,
p=',round(gray.pval[3,2],3)),cex=1.5)

text(max.x-10,0.6,paste0('DM                                     only,
p=',round(gray.pval[2,2],3)),cex=1.5)

text(max.x-10,0.9,'Disease-Free',cex=1.5)



# save CIF for PORT

cif.p <- cif; cif.p.lr <- cif.1; cif.p.dm <- cif.2; cif.p.lrdm <- cif.3;
cif.p.d <- cif.4



dev.off()





closeAllConnections() # Close connection to log file
 Obs PORT
 180  184

  Male Female
   202    162

Age <=60 years  Age >60 years
           271             93

No Smoking    Smoking
       202        162

KPS >=90  KPS <90
     177      187

 Left Right
  144   220
```

```
 No Yes 
123 241 

NonSquamous    Squamous 
        304          60 

postop dmax<=3cm  postop dmax>3cm 
             190              174 

   Path T1 Path T2-T3 
        81        283 

Lymph Nodes <=20 Lymph Nodes > 20 
             172              192 

Pos Lymph Nodes <= 3   Pos Lymph Nodes >3 
                 153                  211 

    EGFR Sensitive EGFR non-Sensitive 
               219                145 

  0   1   2 
211 144   9 

  0   1   2   3   4 
134  51 139  36   4 

  0   1 
 54 310 

  0   1 
214 150 
$quantile
      25       50       75 
27.00616 46.02875 71.52361 

$lower
      25       50       75 
24.87064 41.88912 63.50719 

$upper
      25       50       75 
30.19302 51.35113 76.18891 
```

```
Call:
coxph(formula = Surv(DFS_months, DFS_status) ~ trt, data = port)

  n= 364, number of events= 230

           coef exp(coef) se(coef)      z Pr(>|z|)
trtPORT -0.1693    0.8442   0.1321 -1.282      0.2

        exp(coef) exp(-coef) lower .95 upper .95
trtPORT    0.8442      1.185    0.6516     1.094

Concordance= 0.52  (se = 0.018 )
Likelihood ratio test= 1.64  on 1 df,   p=0.2
Wald test            = 1.64  on 1 df,   p=0.2
Score (logrank) test = 1.65  on 1 df,   p=0.2

[1] 0.09965944
Call:
coxph(formula = Surv(OS_months, OS_status) ~ trt, data = port)

  n= 364, number of events= 97

           coef exp(coef) se(coef)     z Pr(>|z|)
trtPORT 0.01696   1.01710  0.20357 0.083    0.934

        exp(coef) exp(-coef) lower .95 upper .95
trtPORT     1.017     0.9832    0.6825     1.516

Concordance= 0.516  (se = 0.028 )
Likelihood ratio test= 0.01  on 1 df,   p=0.9
Wald test            = 0.01  on 1 df,   p=0.9
Score (logrank) test = 0.01  on 1 df,   p=0.9

[1] 0.9342387
Call:
coxph(formula = Surv(LRFS_months, LRFS_status) ~ trt, data = port)

  n= 364, number of events= 151

           coef exp(coef) se(coef)     z Pr(>|z|)
trtPORT -0.3496    0.7050   0.1641 -2.13   0.0332 *
---
Signif. codes:  0 '***' 0.001 '**' 0.01 '*' 0.05 '.' 0.1 ' ' 1
```

```
        exp(coef) exp(-coef) lower .95 upper .95
trtPORT     0.705      1.418    0.5111    0.9725

Concordance= 0.534  (se = 0.022 )
Likelihood ratio test= 4.57  on 1 df,   p=0.03
Wald test            = 4.54  on 1 df,   p=0.03
Score (logrank) test = 4.58  on 1 df,   p=0.03

[1] 0.03224053
Call:
coxph(formula = Surv(DMFS_months, DMFS_status) ~ trt, data = port)

  n= 364, number of events= 215

            coef exp(coef) se(coef)      z Pr(>|z|)
trtPORT -0.06739   0.93483  0.13654 -0.494    0.622

        exp(coef) exp(-coef) lower .95 upper .95
trtPORT    0.9348       1.07    0.7153     1.222

Concordance= 0.503  (se = 0.018 )
Likelihood ratio test= 0.24  on 1 df,   p=0.6
Wald test            = 0.24  on 1 df,   p=0.6
Score (logrank) test = 0.24  on 1 df,   p=0.6

[1] 0.6211104
Call:
coxph(formula = Surv(DFS_months, DFS_status) ~ trt, data = port.pp)

  n= 310, number of events= 196

           coef exp(coef) se(coef)     z Pr(>|z|)
trtPORT -0.2843    0.7525   0.1451 -1.96     0.05 *
---
Signif. codes:  0 ‘***’ 0.001 ‘**’ 0.01 ‘*’ 0.05 ‘.’ 0.1 ‘ ’ 1

        exp(coef) exp(-coef) lower .95 upper .95
trtPORT    0.7525      1.329    0.5663         1

Concordance= 0.536  (se = 0.019 )
Likelihood ratio test= 3.89  on 1 df,   p=0.05
Wald test            = 3.84  on 1 df,   p=0.05
Score (logrank) test = 3.87  on 1 df,   p=0.05
```

```
[1] 0.04907455
Call:
coxph(formula = Surv(OS_months, OS_status) ~ trt, data = port.pp)

  n= 310, number of events= 77

           coef exp(coef) se(coef)      z Pr(>|z|)
trtPORT -0.1912    0.8260   0.2307 -0.829    0.407

        exp(coef) exp(-coef) lower .95 upper .95
trtPORT     0.826      1.211    0.5256     1.298

Concordance= 0.515  (se = 0.031 )
Likelihood ratio test= 0.69  on 1 df,   p=0.4
Wald test            = 0.69  on 1 df,   p=0.4
Score (logrank) test = 0.69  on 1 df,   p=0.4

[1] 0.4058983
Call:
coxph(formula = Surv(LRFS_months, LRFS_status) ~ trt, data = port.pp)

  n= 310, number of events= 126

           coef exp(coef) se(coef)      z Pr(>|z|)
trtPORT -0.5874    0.5558   0.1864 -3.151  0.00163 **
---
Signif. codes:  0 ‘***’ 0.001 ‘**’ 0.01 ‘*’ 0.05 ‘.’ 0.1 ‘ ’ 1

        exp(coef) exp(-coef) lower .95 upper .95
trtPORT    0.5558      1.799    0.3857    0.8009

Concordance= 0.567  (se = 0.023 )
Likelihood ratio test= 10.36  on 1 df,   p=0.001
Wald test            = 9.93  on 1 df,   p=0.002
Score (logrank) test = 10.21  on 1 df,   p=0.001

[1] 0.001388393
Call:
coxph(formula = Surv(DMFS_months, DMFS_status) ~ trt, data = port.pp)

  n= 310, number of events= 183

           coef exp(coef) se(coef)     z Pr(>|z|)
trtPORT -0.1628    0.8498   0.1493 -1.09    0.276
```

```
        exp(coef) exp(-coef) lower .95 upper .95
trtPORT    0.8498      1.177    0.6341     1.139

Concordance= 0.517  (se = 0.02 )
Likelihood ratio test= 1.2  on 1 df,   p=0.3
Wald test            = 1.19  on 1 df,   p=0.3
Score (logrank) test = 1.19  on 1 df,   p=0.3

[1] 0.2745708
Call:
coxph(formula = Surv(DFS_months, DFS_status) ~ at, data = port)

  n= 364, number of events= 230 

      coef exp(coef) se(coef)     z Pr(>|z|)  
at -0.3104    0.7332   0.1361 -2.28   0.0226 *
---
Signif. codes:  0 ‘***’ 0.001 ‘**’ 0.01 ‘*’ 0.05 ‘.’ 0.1 ‘ ’ 1

   exp(coef) exp(-coef) lower .95 upper .95
at    0.7332      1.364    0.5615    0.9573

Concordance= 0.541  (se = 0.017 )
Likelihood ratio test= 5.31  on 1 df,   p=0.02
Wald test            = 5.2  on 1 df,   p=0.02
Score (logrank) test = 5.24  on 1 df,   p=0.02

[1] 0.02202327
Call:
coxph(formula = Surv(OS_months, OS_status) ~ at, data = port)

  n= 364, number of events= 97 

      coef exp(coef) se(coef)      z Pr(>|z|)
at -0.3283    0.7201   0.2107 -1.558    0.119

   exp(coef) exp(-coef) lower .95 upper .95
at    0.7201      1.389    0.4765     1.088

Concordance= 0.537  (se = 0.026 )
Likelihood ratio test= 2.49  on 1 df,   p=0.1
Wald test            = 2.43  on 1 df,   p=0.1
Score (logrank) test = 2.45  on 1 df,   p=0.1
```

```
[1] 0.1173716
Call:
coxph(formula = Surv(LRFS_months, LRFS_status) ~ at, data = port)

  n= 364, number of events= 151

      coef exp(coef) se(coef)      z Pr(>|z|)
at -0.6420    0.5262   0.1754 -3.661 0.000251 ***
---
Signif. codes:  0 ‘***’ 0.001 ‘**’ 0.01 ‘*’ 0.05 ‘.’ 0.1 ‘ ’ 1

   exp(coef) exp(-coef) lower .95 upper .95
at    0.5262        1.9    0.3732    0.7421

Concordance= 0.576  (se = 0.02 )
Likelihood ratio test= 14.27  on 1 df,   p=2e-04
Wald test            = 13.4  on 1 df,   p=3e-04
Score (logrank) test = 13.86  on 1 df,   p=2e-04

[1] 0.0001965475
Call:
coxph(formula = Surv(DMFS_months, DMFS_status) ~ at, data = port)

  n= 364, number of events= 215

      coef exp(coef) se(coef)      z Pr(>|z|)
at -0.1996    0.8190   0.1398 -1.428    0.153

   exp(coef) exp(-coef) lower .95 upper .95
at     0.819      1.221    0.6228     1.077

Concordance= 0.524  (se = 0.018 )
Likelihood ratio test= 2.07  on 1 df,   p=0.2
Wald test            = 2.04  on 1 df,   p=0.2
Score (logrank) test = 2.05  on 1 df,   p=0.2

[1] 0.152303
[1] 0.0185568
Call:
coxph(formula = Surv(DFS_months, DFS_status) ~ trt + strata(detected.LN.groups,
    LNM.groups3), data = port)
```

```
  n= 364, number of events= 230

           coef exp(coef) se(coef)      z Pr(>|z|)
trtPORT -0.2836    0.7530   0.1365 -2.078   0.0377 *
---
Signif. codes:  0 '***' 0.001 '**' 0.01 '*' 0.05 '.' 0.1 ' ' 1

        exp(coef) exp(-coef) lower .95 upper .95
trtPORT     0.753      1.328    0.5762    0.9841

Concordance= 0.533  (se = 0.018 )
Likelihood ratio test= 4.32  on 1 df,   p=0.04
Wald test            = 4.32  on 1 df,   p=0.04
Score (logrank) test = 4.34  on 1 df,   p=0.04

[1] 0.6980555
Call:
coxph(formula = Surv(OS_months, OS_status) ~ trt + strata(detected.LN.groups,
    LNM.groups3), data = port)

  n= 364, number of events= 97

            coef exp(coef) se(coef)      z Pr(>|z|)
trtPORT -0.08115   0.92206  0.20923 -0.388    0.698

        exp(coef) exp(-coef) lower .95 upper .95
trtPORT    0.9221      1.085    0.6119     1.389

Concordance= 0.503  (se = 0.029 )
Likelihood ratio test= 0.15  on 1 df,   p=0.7
Wald test            = 0.15  on 1 df,   p=0.7
Score (logrank) test = 0.15  on 1 df,   p=0.7
```
