# Supplementary material for: Effect of Postoperative Radiotherapy for Patients With pIIIA-N2 Non–Small Cell Lung Cancer After Complete Resection and Adjuvant Chemotherapy: The Phase 3 PORT-C Randomized Clinical Trial
Source: JAMA Oncol. 2021 Jun 24;7(8):1–8. doi: 10.1001/jamaoncol.2021.1910 (PMC8227450; doi:10.1001/jamaoncol.2021.1910)
Supplement: Supplement 2. — eAppendix 1. Diagram eAppendix 2. RT parameters [file jamaoncol-e211910-s002.pdf]

Supplemental Online Content

Hui Z, Men Y, Hu C, et al. Effect of postoperative radiotherapy for patients with pIIIA-N2 non–small cell lung cancer after complete resection and adjuvant chemotherapy: the phase 3 PORT-C randomized clinical trial. *JAMA Oncol*. Published online June 24, 2021. doi:10.1001/jamaoncol.2021.1910

eAppendix 1. Diagram

eAppendix 2. RT parameters

This supplemental material has been provided by the authors to give readers additional information about their work.

© 2021 Hui Z et al. *JAMA Oncology.*

eAppendix 1. Diagram

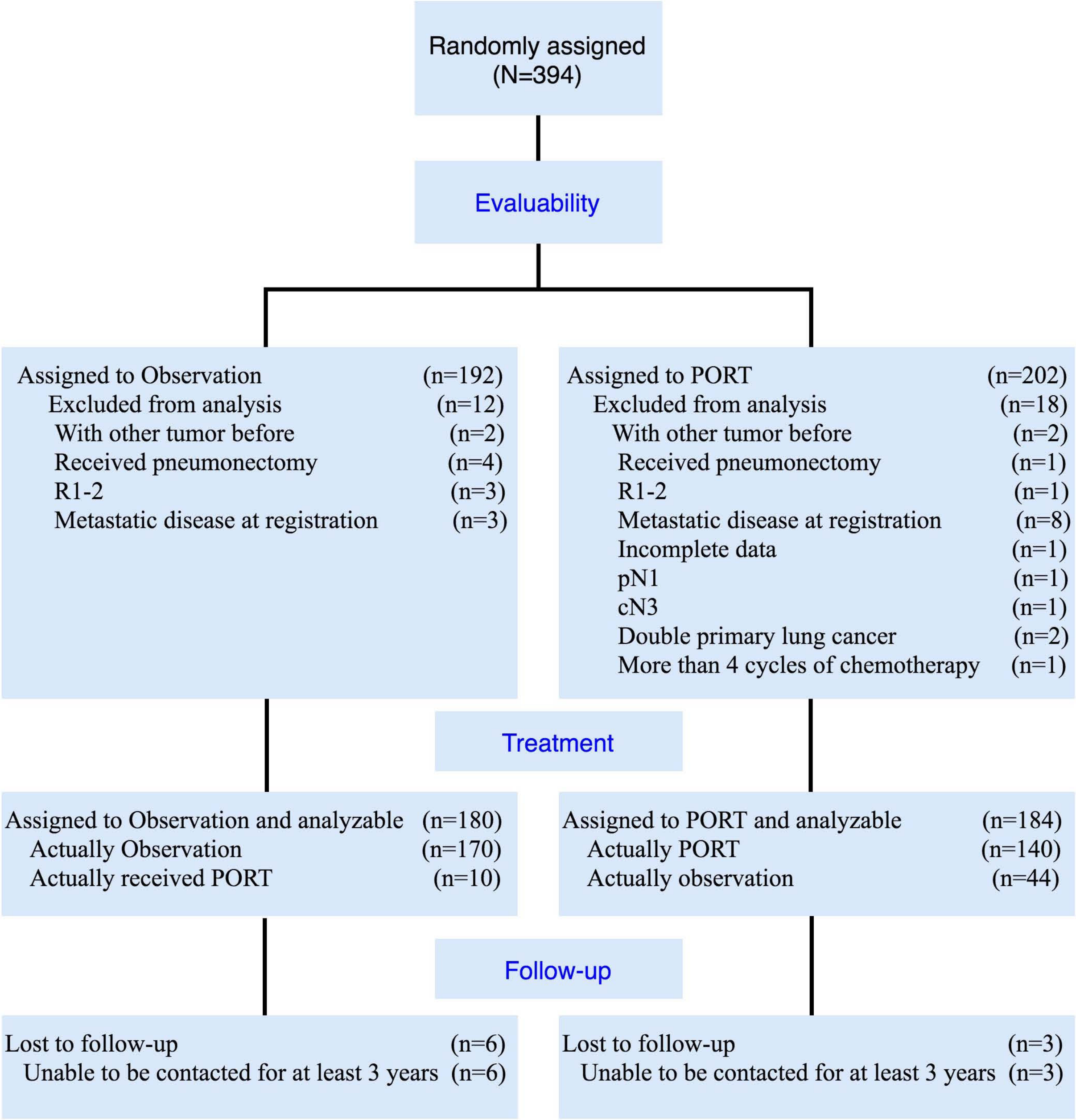

© 2021 Hui Z et al. *JAMA Oncology.*

eAppendix 2. OARs dosimetric parameters in patients receiving PORT

**Parameters**	**Dose constraints**	**Actually delivered**	
		**Dose (median)**	**Dose (mean ± SD)**
Lung			
V20 (%)	＜25	16.73	16.47±3.46
MLD (Gy)	＜12	9.63	10.44±8.22
Spinal Cord			
Dmax (Gy)	＜45	33.72	34.12±4.91
Heart			
V30 (%)	＜40	10.44	12.16±9.94
V40 (%)	＜30	4.81	6.29±6.04

Abbreviations: OAR: Organs at risks; V20 to V40: Percentage of the organ receiving more than a specific dose of radiation; MLD: Mean lung dose.

© 2021 Hui Z et al. *JAMA Oncology.*
