# Supplementary material for: Effect of Postoperative Radiotherapy for Patients With pIIIA-N2 Non–Small Cell Lung Cancer After Complete Resection and Adjuvant Chemotherapy: The Phase 3 PORT-C Randomized Clinical Trial
Source: JAMA Oncol. 2021 Jun 24;7(8):1–8. doi: 10.1001/jamaoncol.2021.1910 (PMC8227450; doi:10.1001/jamaoncol.2021.1910)
Supplement: Supplement 3. — Data Sharing Statement [file jamaoncol-e211910-s003.pdf]

# Data Sharing Statement

Hui. Effect of Postoperative Radiotherapy for Patients With pIIIA-N2 Non-Small Cell Lung Cancer After Complete Resection and Adjuvant Chemotherapy. *JAMA Oncol*. Published June 24, 2021.
doi:10.1001/jamaoncol.2021.1910

**Data**
**Data available:** No
